# Erratum: Rethinking disability: The need to rethink representation

**DOI:** 10.4102/ajod.v8i0.605

**Published:** 2019-10-10

**Authors:** Jenna-Lee Procter

**Affiliations:** 1 Department of Psychology, Stellenbosch University, South Africa

In the version of this article published earlier, the term ‘mixed race’ was unintentionally misused and is hereby updated to be ‘people of colour’.

The term ‘People of colour’ includes people of *all* non-white racial or ethnic groups in the world that have been and are still being disenfranchised or marginalised by white privilege. ‘People of colour’ should not be confused with the South African term ‘coloured’ (as per the *Employment Equity Act* No. 55 of 1998). ‘People of colour’ is by definition a world-wide anti-racist identity.

The paragraph is hereby updated to read as follows:

I was disappointed to discover, despite emphasising on disABILITY MUNDUS, that the volume’s dominant voice came from the white, global North. Only a few of the contributors were people of colour and none, as far as I could tell, were from Africa, Central or East Asia, India or Eastern Europe. I found this lack of representation problematic in a text that espouses ‘world perspectives’ and uses a title which implies that ‘world’ is synonymous with North America and Western Europe. The exclusion of African researchers from the global academy is indicative of the failure to decolonise, losing out on useful transnational and transferable identity capital. The majority of people with disabilities in fact live in the global South, and we have many competent, ardent and inspiring disability scholars and academics who could have contributed to *Rethinking Disability.*

The publisher apologises for any inconvenience that the usage of the incorrect term may have caused.

